# Organoids for toxicology and genetic toxicology: applications with drugs and prospects for environmental carcinogenesis

**DOI:** 10.1093/mutage/geab023

**Published:** 2021-06-19

**Authors:** Angela L Caipa Garcia, Volker M Arlt, David H Phillips

**Affiliations:** Department of Analytical, Environmental and Forensic Sciences, School of Population Health and Environmental Sciences, King’s College London, London, SE1 9NH, UK

## Abstract

Advances in three-dimensional (3D) cell culture technology have led to the development of more biologically and physiologically relevant models to study organ development, disease, toxicology and drug screening. Organoids have been derived from many mammalian tissues, both normal and tumour, from adult stem cells and from pluripotent stem cells. Tissue organoids can retain many of the cell types and much of the structure and function of the organ of origin. Organoids derived from pluripotent stem cells display increased complexity compared with organoids derived from adult stem cells. It has been shown that organoids express many functional xenobiotic-metabolising enzymes including cytochrome P450s (CYPs). This has benefitted the drug development field in facilitating pre-clinical testing of more personalised treatments and in developing large toxicity and efficacy screens for a range of compounds. In the field of environmental and genetic toxicology, treatment of organoids with various compounds has generated responses that are close to those obtained in primary tissues and *in vivo* models, demonstrating the biological relevance of these *in vitro* multicellular 3D systems. Toxicological investigations of compounds in different tissue organoids have produced promising results indicating that organoids will refine future studies on the effects of environmental exposures and carcinogenic risk to humans. With further development and standardised procedures, advancing our understanding on the metabolic capabilities of organoids will help to validate their use to investigate the modes of action of environmental carcinogens.

## Introduction

**Fig. 1.**
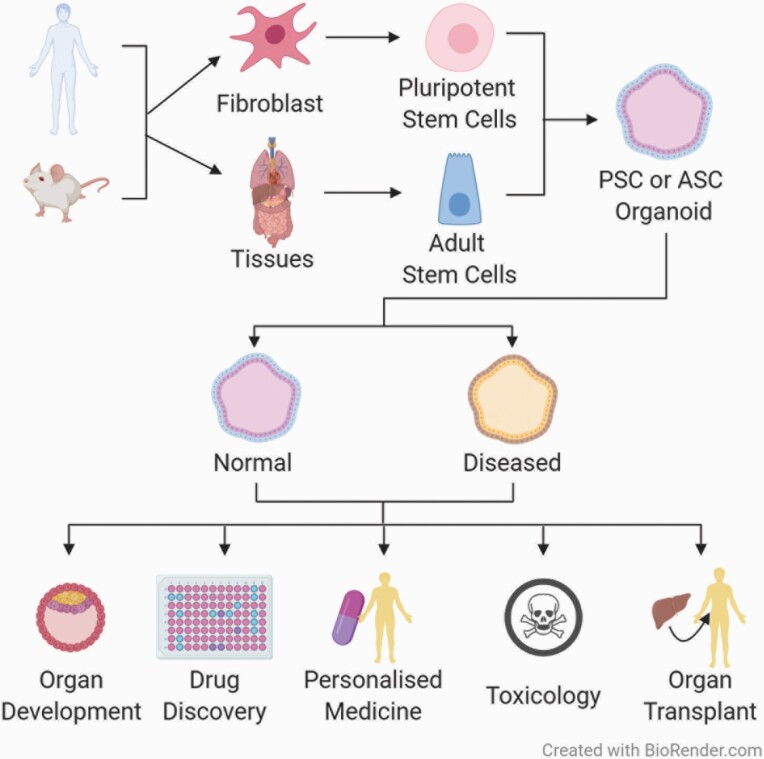
Scheme showing the origin and application of organoid systems. Organoids can be derived from human and animal (e.g. mouse) pluripotent or adult stem cells. Normal and diseased organoids can be used for many applications such as disease modelling, organ development, drug screening, personalised medicine, toxicology and organ transplant and replacement.

Advances in three-dimensional (3D) cell culture technology in recent years have increased its use in several fields including organ development, disease modelling and drug screening. A number of 3D models have been established, from simple unicellular spheroid cultures to more complex systems, like organoids and microphysiological systems (MPS) (1–3). Organoids are multicellular 3D cultures derived from stem cells that self-assemble into structures that contain organ-specific cell types and that recreate some of the *in vivo* cell organisation and functions of the organ of origin (4). As shown in Figure 1, organoids can originate from pluripotent stem cells (PSCs), both embryonic and induced pluripotent stem cells (iPSCs), or adult stem cells (ASCs), which can proliferate indefinitely in culture and have been shown to have the ability to differentiate and re-organise to mimic their source organs (5).

The derivation process of PSC organoids exploits both the ability of these stem cells to differentiate into several cell types and the organ development process, including spatial patterning and morphogenesis (5–8). The ability to generate organoids from ASCs started with the identification of the stem cell marker leucine-rich repeat containing G-protein-coupled receptor 5 (*Lgr5*) in intestinal epithelium, which allowed the characterisation of multipotent and self-renewing adult stem cell populations in several other tissues, including colon (9), stomach (10), pancreas (11) and liver (12). These *Lgr5*^*+*^ ASCs, from isolated cells or from dissected tissue fragments, can be used to establish organoids by providing an adequate environment to mimic tissue homeostasis and regeneration (5,10,12,13).

The use of human organoids has become increasingly widespread over the past few years as alternatives to more routinely used model systems, such as animals and two-dimensional (2D) mammalian cell cultures. The perceived need for more physiologically relevant *in vitro* models has been the main reason for this shift towards the use of 3D cultures in different research fields (14–18). Although experimental animals, mainly rodents, have provided substantial amounts of information about many biological processes and have been a key resource for chemical and drug safety studies, these animal models do not always accurately represent the human response due to species-specific disease states and reactions (19–21). Similarly, 2D mammalian cell cultures have been widely used in research for more than a hundred years and have led to the discovery of countless pathways and processes in biology. In spite of this, the dependability of results from studies using 2D cultures for drug and chemical safety and efficacy can been questioned due to the highly artificial nature of their culture environment (22,23). Therefore, the use of human organoids and other 3D cultures may allow the collection of data more relevant to human physiology while contributing to reductions in the use of animals in basic and applied research (24–28).

As mentioned earlier, the application of human organoid systems includes, but is not limited to, organ development, disease modelling and pharmacological studies (Figure 1). PSC-derived human organoids have proved to be very useful for studying organ development, where their propensity to reproduce embryonic stages of development has allowed the replication of key steps in organogenesis, such as spatial organisation of the heart, the brain, the gastrointestinal tract and other organs (29–32). The use of human organoids in disease modelling has led to the establishment of assays and models that assist with diagnosis, drug screening and personalised treatment. This has been achieved for a number of conditions like cystic fibrosis, for which tubuloids from urine, as well as intestinal and airway organoids from patients, have been used to identify treatments that benefit patients with specific mutations (18,33,34). Progress has also been made in cancer research, using biobanked samples of human tumour and normal organoids derived from cancer patients to better understand tumour heterogeneity and responses to chemotherapy, as well as aiding the improvement of personalised therapy (35–38). Drug screening using human organoids is already being carried out to facilitate pre-clinical drug development, as well as to predict an individual patient’s response to treatment (17,18,37). It is anticipated that this will not only maximise clinical benefits of existing therapies, but also enable increased adoption of novel therapeutics (39). Engraftment of organoids in mice has demonstrated the use of organoids in regenerative medicine and transplantation is also very promising, as it appears to minimise the risks of transplant rejection and increase the availability of healthy tissue (40).

Additionally, organoids show great potential in drug toxicology studies (41–46). However, their use in environmental and genetic toxicology is at a relatively early stage. This review will focus on the derivation of organoids and their use in environmental and genetic toxicology, detailing the studies that have been conducted and considering the future potential of organoids in this important field.

## Organoid Models: iPSC vs ASC Derived Organoids

Organoid models are characterised by the self-organisation of cells in culture into *in vivo*-like structures; however, their derivation depends on the starting material. Organoids from both embryonic and induced pluripotent stem cells have been established from several organs, including gut, kidney, liver, lung, intestine and brain (47). Derivation of these PSC organoids utilises knowledge of the cell sorting and lineage commitment pathways, combined with growth in culture under conditions specific for the desired differentiation pathway (4). This process usually takes a few weeks to generate mature organoids (6). In the case of ASC-derived organoids, Wnt signalling pathway activation is key for their establishment (48). These organoids, which originate from *Lgr5*^*+*^ stem cells obtained from single cell sorting or dissected tissue fragments, are grown in organ-specific culture media that contains Wnt activators such as R-spondin 1 and, in some cases, Wnt3A (48). Organoid types that have been derived from ASCs include gut, liver, pancreas, intestine, kidney and also from mammary and salivary glands (47). In contrast to PSC-derived organoids, the process to generate mature organoids from ASCs takes only a few days (11,13).

### Small intestine organoids

**Fig. 2.**
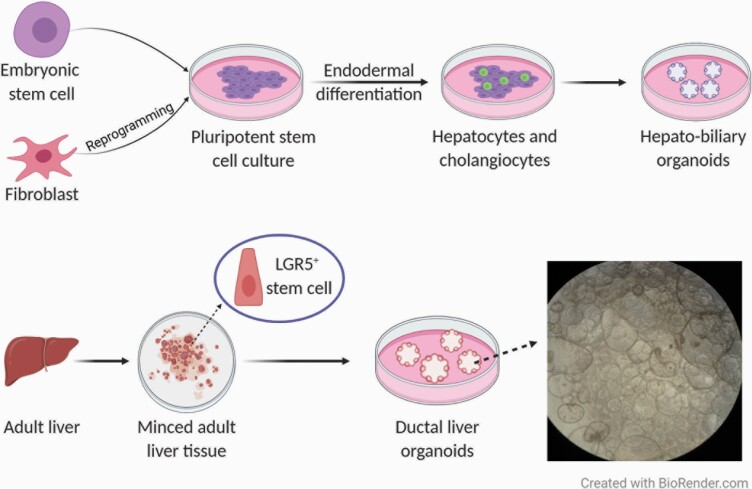
Scheme of liver organoid culture formation from pluripotent stem cells or adult stem cells. On the top panel, pluripotent stem cells, embryonic or induced, undergo differentiation towards the desired cell type. PSCs are differentiated into hepatocytes and cholangiocytes which then generate organoid cultures containing both hepatocytes and bile duct cells. The bottom panel shows how organoids can be derived from minced adult tissue which contains Lgr5^+^ stem cells that in this case can give rise to liver organoid cultures containing ductal cells. Organoid cultures are embedded in basement membrane extract and are grown in media complemented with essential growth factors. Adapted from (66,119). An example of normal human adult liver organoids grown in our lab is shown on the right in the bottom panel.

Although both PSC and ASC-derived organoids can be grown in culture for a long time, they have distinct differences, not only in the stages of development they represent, but also in the cell types they contain, and, therefore, in their complexity (Figure 2). A clear example of this can be seen with gastrointestinal tract organoids (49). Those derived from ASCs contain only organ-specific epithelial and stem cells, while organoids derived from PSCs contain epithelial cells as well as mesenchymal cells, including fibroblasts and smooth muscle, due to the ability of PSCs to differentiate into any cell type (50). Small intestine organoids were first derived from ASCs by the Clevers lab at the Hubrecht Institute, The Netherlands (13). These organoids established from single *Lgr5*^*+*^ cells form crypts containing Paneth cells as well as *Lgr5*^*+*^ stem cells that surround a central lumen lined by a villus-like epithelium with polarised enterocytes, and goblet and enteroendocrine cells dispersed throughout the organoid (13,51). This has been replicated a number of times from murine and human tissues and adapted for the establishment of intestinal organoids from cystic fibrosis patients (34,52,53). Intestinal tissue organoids originating from human PSCs were first reported by Spence *et al.* (6). In this study, embryonic intestinal development was mimicked by using a series of culture conditions that included growth factors to induce intestinal growth, morphogenesis and cytodifferentiation. These organoids contained polarised, columnar epithelium resembling villus- and crypt-like structures with all intestinal endothelial cells present, as well as a layer of mesenchymal cells including subepithelial myofibroblasts, smooth muscle and fibroblasts (6). This model has been replicated and used for different purposes such as generating *in vivo* human organoid engraftment mouse models and viral infection models, amongst others (54,55).

### Colon organoids

As with the small intestine, colon and gut organoids have also been derived both from PSCs and from ASCs. Colon *Lgr5*^*+*^ ASCs were identified in the Clevers lab, where colon organoids were later established from human and murine tissues (9,51). Tissue-derived colonic organoids appeared as cystic or budding structures with proliferative cells; subsequently when culture conditions were modified, goblet and enteroendocrine cells were also present (51,56). Human PSC-derived colon organoids were established by stimulating bone morphogenetic protein (BMP) signalling; these organoids comprised colon-specific goblet and enteroendocrine cells as well as mesenchyme (57). At the same time, others also generated PSC-colonic organoids by using a differentiation method that involved the modulation of the Wnt pathway via the addition of glycogen synthase kinase (GSK)-3β inhibitors as well as BMP regulation (58).

### Gastric organoids

Gut or gastric organoids were first derived from mouse *Lgr5+* ASCs and they resembled the pyloric epithelium organised around a central lumen (10). Human ASC gastric organoids were later established from different regions of the stomach; these consisted of budding or cystic structures comprised of mainly mucous gland, chief and enteroendocrine cells surrounding a central lumen (59,60). These human tissue gastric organoids can also be further differentiated by altering the culture conditions in order to obtain the various gastric cell lineages (59,60). In a similar way, PSC-derived gut organoids formed simple gland and pit domains and contained *Lgr5*^*+*^ cells as well as mucous and endocrine cells; however, they represented early developmental stages and contained submucosal myofibroblasts and a small population of subepithelial myofibroblasts (32).

### Liver organoids

As mentioned earlier, organoids derived from both types of stem cell populations have also been established for the liver, pancreas and kidney (see below for more details on pancreatic and kidney organoids). In adults, *Lgr5* expression in these organs is low under normal conditions, however, after injury, the Wnt pathway is activated leading to increased expression of *Lgr5* and, therefore, tissue regeneration (11,12). ASC-derived liver organoids were first reported by Huch *et al.* (11,12); they consisted of large cysts with *Lgr5*^*+*^ cells and bile duct cells, while hepatocyte markers were only weakly expressed. Hepatocyte maturation was achieved by altering culture conditions; however, this led to a concurrent decrease in the expression of proliferative cells (12,61,62). Hepatocyte-derived organoids were later established from murine and human single mature hepatocytes. They resembled the main functions and gene expression patterns of hepatocytes *in vivo* and could proliferate in culture for at least 6 months (63,64). PSC-derived liver organoids were first generated as vascularised liver buds. These liver organoids originated by co-culturing human hepatic endoderm and stromal cells, expressed early liver-specific markers including alpha-fetoprotein and albumin, and were capable of drug metabolism (65). More recently, hepatobiliary organoids obtained from human PSCs were established. These organoids displayed functions of both hepatocytes (e.g. drug metabolism and albumin production) and bile duct cells (66) (Figure 2). Other investigators have established hepatocyte-like organoids that resemble adult liver tissue-derived organoids (43).

### Pancreatic organoids

Pancreatic organoids were first made from ductal fragments of mouse pancreas. These formed cysts that contained duct cells *in vitro,* some of which differentiated into endocrine cells *in vivo* (11). This protocol was then adapted for the growth of human tissue organoids that also comprised stem and ductal cells (62,67). Human tissue pancreatic organoids that have the potential to differentiate into endocrine lineage cells *in vitro* were later produced from digested islet-depleted pancreatic tissue. Initially these formed budding structures with almost all cells displaying a ductal phenotype with progenitor cells at the tip regions, some of which differentiated into endocrine cells and produced insulin after transplantation (68). Organoids that reproduce pancreatic development were also derived from mouse foetal pancreatic progenitors. These were composed of epithelial cells that expressed progenitor markers such as *Pdx1*, *Sox9*, *Hnf1b* and *Nkx2.2*, which then differentiated into ductal and exocrine lineages (69). Pancreatic organoids from PSCs formed hollow structures surrounded by a layer of polarised epithelium that comprised cells expressing exocrine and progenitor cell markers, as well as cells capable of secreting collagen IV and laminin-α5 (70).

### Kidney organoids

Kidney organoids have been mainly derived from human PSCs and several protocols have been published for the generation of kidney organoids containing different kidney cell types. The formation of kidney organoids from human embryonic stem cells through stepwise differentiation into the key developmental lineages of the kidney was reported (71). These organoids contain ureteric buds and metanephric mesenchyme, including early nephrons, as well as cells expressing podocyte, proximal tubule and collecting duct genes. Others reported deriving organoids from mouse and human stem cells in which proximal and distal tubules were formed, as well as glomerulus-like structures, and cells with podocyte markers were also seen (72). Like these, many other nephron organoids expressing podocyte, proximal tubule, loops of Henle and distal tubule markers with different degrees of differentiation and complexity have been generated from human and murine embryonic and pluripotent stem cells (14,73–76). ASC-derived kidney organoids have been named ‘tubuloids,’ as they mainly contain tubular epithelial cells, and similarly to organoids derived from adult tissues they reproduce tissue regeneration rather than development (77). Tubuloids from normal human adult tissue were reported consisting of cystic structures containing tubular epithelial cells that resembled proximal tubular cells and that expressed the renal tubule protein Tamm-Horsfall (78). Others established tubuloids from human tissues that showed high expression of proximal tubule markers, as well as some collecting duct, loop of Henle and distal tubule markers (33). Tubuloids were also established from cells isolated from urine of cystic fibrosis patients and from kidney tumours (33).

## Organoids in Drug Screening and Toxicology

Due to their structural and functional features, organoid models have great potential in toxicology studies. Table 1 lists studies in which organoids derived from various human and animal tissues from both PSCs and ASCs have been used in toxicological research. Different aspects have been investigated, from the effects and efficiency of drugs to the toxicity of environmental pollutants. The former has been investigated more widely, possibly due to the opportunity that testing of large libraries of therapeutic compounds on patient-derived organoids offers to bridge the gap between monolayer cell culture and animal models, and the relevance of the results for human physiology in pre-clinical trials (46,79).

**Table 1.** Studies using organoids in drug screening and toxicology

Therapeutic drugs			
**Organoid type**	**Aim of the study**	**Key findings**	**Reference**
Oesophageal adenocarcinoma – ASCs	Establishment and characterisation of oesophageal adenocarcinoma organoids. Evaluation of their sensitivity to 24 anticancer compounds, including approved drugs and preclinical targeted agents.	Organoids recapitulated the features of the primary tumour. A range of compound sensitivities, including drug potency and efficacy, was observed, which was consistent with patient responses to therapy (e.g. resistance).	(17)
Ductal pancreatic cancer – ASCs and PSCs	Development of pancreatic tumoroids from PSCs and patient samples that model ductal pancreatic cancer. Assessment of patient tumour organoid responses to gemcitabine and drugs targeting epigenetic regulators.	Patient-derived tumour organoids maintained tumour-specific traits. Treatment with gemcitabine led to poor response with only 30% growth inhibition and treatment with epigenetic regulators decreased proliferation to varied degrees in different organoids.	(70)
Pancreatic tumour – ASCs	Studying human pancreatic tumoroid survival in co-culture with liver organoids after treatment with docetaxel.	Pancreatic tumoroid co-culture with CYP-induced liver organoids showed higher survival rate compared with undifferentiated and differentiated liver after treatment with docetaxel.	(46)
Colon tumour and normal – ASCs	Establishment and characterisation of colon tumour and normal organoid lines. Assessment of organoids for their sensitivity after treatment with a library of 83 compounds including chemotherapeutics, drugs in clinical use or in clinical trials and experimental compounds.	Colon tumour organoids recapitulated several features of the primary tumour and reflected the heterogeneity of the tumours. Differential responses of the organoids to the compounds were seen measured by the IC_50_, the slope of the dose–response curve and the area under the curve, as well as organoid-drug interactions.	(37)
Rectal – ASCs	Measurement of drug efficacy of the CFTR-modulating treatments (ivacaftor, lumacaftor plus ivacaftor and genistein plus curcumin) in rectal organoids from cystic fibrosis patients and correlation with *in vivo* effects.	*In vitro* responses to CFTR modulators (ivacaftor, lumacaftor plus ivacaftor and genistein plus curcumin) and forskolin-induced swelling in the rectal organoids from cystic fibrosis patients correlated with two indicators of therapeutic response *in vivo*, sweat chloride concentration and pulmonary response.	(39)
Rectal – ASCs	Studying CFTR function of rectal organoids from cystic fibrosis patients and the organoid’s response to the drug treatments ivacaftor and lumacaftor.	CFTR function and response of rectal organoids to treatment with ivacaftor and lumacaftor depended on the CFTR mutation and the genetic background of the patient. Results positively correlated with published data from clinical trials.	(84)
Kidney cancer – ASCs	Establishment of kidney organoid biobank from paediatric cancer patients. Characterisation of tumour kidney organoids and testing their sensitivity towards drugs used in standard chemotherapy.	Kidney tumour organoids maintain key features of the tumour of origin, including their heterogeneity. Tumour organoid lines were more sensitive, based on a dose–response curve, to some therapies than normal kidney organoids.	(35)
Kidney – PSCs	Establishment of kidney organoids and testing their ability to endocytose dextran and to undergo apoptosis in response to cisplatin treatment.	Demonstration of kidney organoid functionality by selective uptake of dextran. The nephrotoxicant cisplatin induced acute apoptosis in kidney organoids.	(76)
Kidney – PSCs	Establishment of kidney organoids and assessment of drug nephrotoxicity by treating them with gentamicin or cisplatin, drugs known to cause proximal tubular toxicity.	Nephrotoxicity was observed after organoid treatment with these drugs, as proximal and distal tubule biomarkers for toxicity, like kidney injury molecule-1, were expressed.	(14)
Bile tract carcinoma – ASCs	Testing a library of drugs used clinically for their ability to suppress the tumoroids derived from intrahepatic cholangiocarcinoma, gall- bladder cancer and neuroendocrine carcinoma of the ampulla of Vater.	The library screening showed that the antifungal drugs amorolfine and fenticonazole suppress tumour organoid growth while little cytotoxicity was seen in normal cells.	(45)
Liver cancer –ASCs	Studying the functional heterogeneity of liver cancer organoids by testing 129 cancer drugs to assess liver cancer heterogeneity.	Liver cancer organoids showed large variability in intra-tumour drug response, with many drugs showing no cytotoxic response and some drugs effective only in certain organoids. A group of drugs used for other cancers rather than liver cancer showed moderate activity in most organoids.	(36)
Testicular – ASCs	Evaluation of testicular organoids as a model for reproductive toxicity by treatment with cisplatin, etoposide, doxorubicin and busulfan. Comparison of results to those obtained in 2D culture of the same cell types.	The testicular organoids showed a dose-dependent response in terms of cell viability after treatment with cisplatin, etoposide, doxorubicin and busulfan and had IC_50_ values higher than those in the same cell types cultured in 2D.	(41)
Intestinal – ASCs	Studying toxicity and cell death induction in normal intestinal organoids after exposure to cisplatin, 5-fluorouracil, UV or X-ray radiation. Comparison of results to those obtained in colon carcinoma cell lines.	Intestinal organoids were more sensitive to chemotherapeutic drugs than colon carcinoma cell lines, mimicking the *in vivo* situation. The organoids also responded much more sensitively to radiation exposure than the immortal cell lines.	(42)
Intestinal crypt – ASCs	Studying gene expression of XMEs and activation of xenobiotic nuclear receptors in intestinal organoids after treatment with the anticancer pro-drug camptothecin-11.	Expression of XMEs, such as CYPs, Adh1, Ces, Ugts and Sult and transporters, as well as functional nuclear receptors, like the aryl hydrocarbon receptor, were observed. Organoids metabolised the anticancer prodrug camptothecin-11 by the action of Ces and Ugt1a1.	(44)
Liver – PSCs	1) Characterisation of human PSCs-derived liver organoids and functionality assessment. Study of their toxicity profiles after treatment with compounds such as omeprazole, hepatotoxic compounds and antibiotics, and comparison to a 2D model. 2) Development of a steatosis pathology model and testing L-carnitine, metformin and other compounds for their toxicity and efficiency to treat this condition.	1) Human PSCs-derived liver organoids maintain liver properties including the ability to metabolise drugs and showed toxic responses to drug treatment. Organoids were more sensitive than 2D hepatocyte monolayers at clinically relevant doses. 2) Human PSCs-derived liver organoids displayed steatosis phenotypes after treatment with lipids. Treatment with L-carnitine ameliorated the disease phenotype and further compounds were identified from an autophagy library.	(43)
Liver, cardiac, lung, brain, testes and colon – PSCs	Treatment of liver, cardiac, lung, brain, testes and colon organoids with 10 drugs that were recalled due to adverse effects, including troglitazone, astemizole and bromfenac, in comparison to a number of non-toxic drugs (aspirin, ibuprofen, loratadine, ascorbic acid and quercetin) used as control. Treatment of liver and cardiac organoids was conducted individually and together with the other 4 organoid types in a ‘body-on-chip’ system. Comparison of results obtained with a 2D model.	Toxic concentrations were lower in the organoids compared with those in 2D primary cells and immortalised cell lines. However, organoids were more sensitive to some drugs like astemizole and more resistant to others like rofecoxib. Results from the cell lines were less significant as they showed greater variability. Most drugs considered as non-toxic did not show any toxicity in organoids. Results from the ‘body-on-chip’ system showed that when organoids are used in combination on a chip, one organoid type influences the activity of another organoid type creating more complex responses.	(116)
**Environmental and experimental toxicants**			
Colon – ASCs	Investigating the effect of ethanol exposure in normal colon organoids on gene expression and chromatin accessibility.	Identification of more than 1500 differentially expressed genes and 2000 differentially accessible chromatin regions in normal colon organoids after ethanol treatment.	(87)
Liver and cardiac – PSCs	Viability and cytotoxicity assessment of liver and cardiac organoids after exposure to the environmental pollutants lead, mercury, thallium and glyphosate.	Liver and cardiac organoids showed toxicity after treatment with environmental pollutants (lead, mercury, thallium and glyphosate) with thallium being the most toxic compound tested. All pollutants led to a decrease in cardiac organoid beating activity.	(15)
Mammary – ASCs	Studying the effect on organoid formation and morphology in organoids derived from mammoplasty patients treated with physiologically relevant doses of cadmium.	Cadmium negatively affected mammary organoid formation and branching at the highest concentration tested, 2.5 µM. Gene expression analysis showed the up- regulation of metal response genes, like metallothioneins and zinc transporters, and inhibited hypoxia inducible factor-1α activity.	(86)
Brain (organoid on chip) – PSCs	Assessment of the effects of nicotine in brain organoids on a chip that recapitulated features of the developing foetal human brain at early stages.	Organoids exposed to nicotine showed premature and abnormal neuronal differentiation. Brain regionalisation and development were also affected.	(106)
Intestinal – ASCs	Studying toxicity and cell death induction in normal intestinal organoids after exposure to cisplatin, 5-fluorouracil, UV or X-ray radiation. Comparison of results to those obtained in colon carcinoma cell lines.	Intestinal organoids were more sensitive to chemotherapeutic drugs than colon carcinoma cell lines, mimicking the *in vivo* situation. The organoids also responded much more sensitively to radiation exposure than the immortal cell lines.	(42)
Small intestine and liver – ASCs	Assessment of drug-metabolising enzymes and evaluation of CYP induction after treatment of organoids with the CYP inducers dexamethasone, β- naphthoflavone and 1,4-bis-2-(3,5-dichloropyridyloxy)-benzene in murine intestinal and liver organoids.	Expression of *CYP1A1, CYP1A2, CYP2A12, CYP2C37, CYP3A11* and *CYP3A13* in both intestinal and liver organoids. These CYPs were differentially induced indicating high drug-metabolic capacity.	(46)
Kidney – ASCs	Toxicity assessment of hydroxylated generation-5 PAMAM dendrimer (G5- OH) nanoparticles and gold nanoparticles in kidney organoids. Comparison to *in vivo* models.	PAMAM nanoparticles induced toxicity biomarkers like Kim-1, neutrophil gelatinase- associated lipocalin, osteopontin, clusterin, vimentin, haem oxygenase-1 and cell toxicity in kidney organoids, mirroring previously published *in vivo* data.	(117)
Lung, liver and mammary –ASCs	Assessment of the *in vivo* tumorigenicity of organoids after *in vitro* treatment with ethyl methanesulfonate, acrylamide, diethylnitrosamine and 7,12-dimethylbenz[*a*]anthracene.	Lung, liver and mammary organoids treated *in vitro* with ethyl methanesulfonate, acrylamide, diethylnitrosamine and 7,12-dimethylbenz[*a*]anthracene were injected into mice leading to the formation of subcutaneous nodules *in vivo,* indicating the carcinogenic potential of the chemicals tested.	(16)
Mammary – ASCs	Studying the effects of bisphenol A, mono-*n*-butyl phthalate and polychlorinated biphenyl 153 on the proteome in mammary organoids.	Treatment of mammary organoids with bisphenol A, mono-*n*-butyl phthalate and polychlorinated biphenyl 153 induced differential effects on the proteome. Treatment also altered the abundance of protein splice variants.	(85)
Endometrial –ASCs	Characterisation of the impact of zinc stearate (plastic additive) on the development of organoids from endometrial cells from domestic cats.	Zinc stearate did not affect morphology, viability or cellular composition of endometrial organoids. The model developed could be used in future studies investigating the effects of plastic additives or drugs.	(118)

### Metabolic potential of organoids

A few studies have focused on investigating the xenobiotic metabolic potential of organoids, primarily in liver and intestinal organoids. These studies have shown that the expression and function of enzymes responsible for the biotransformation of xenobiotic compounds, e.g. cytochrome P450s (CYP), are maintained in the organoids (44,46,61,66,80). CYP3A4 is one of the main xenobiotic-metabolising enzymes (XMEs) in liver and intestine (81). The expression of this enzyme in differentiated liver organoids, i.e. hepatocyte-like organoids, has been shown to be at levels slightly lower than those in adult liver but significantly higher than those seen in foetal liver and stem cells (61,66). The activity of CYP3A4 has also been shown to be significantly higher in differentiated liver organoids than in commonly used cell lines such as human hepatoma HepG2 cells (61,66). Levels of expression of other CYP enzymes important in drug metabolism were compared in mouse small intestine and liver organoids with the respective tissues (46). These investigations showed that mouse *Cyp1a2* and *Cyp3a11* mRNA levels were higher in intestinal organoids than those in the tissue, whilst only *Cyp1a2* mRNA was greater in the liver organoids than in the tissue (46). The ability of organoids to metabolise drugs was also investigated in mouse crypt organoids, which express both Phase I and Phase II XMEs (44). In the latter study, organoid toxicity was also seen after treatment with camptothecin-11, demonstrating that crypt organoids can metabolise this compound. Furthermore, data from our group show a high induction of *CYP1A1* mRNA in human pancreatic, gastric and liver organoids after treatment with the environmental carcinogen benzo[*a*]pyrene (BaP) (Caipa Garcia *et al.*, unpublished results). Collectively, these studies illustrate the utility of organoids for investigations of xenobiotic metabolism and toxicity.

### Disease modelling

The use of organoids for disease modelling could provide a very valuable tool in many areas including drug screening and personalised therapy (82). The availability of organoids derived from patient samples has allowed research on these areas by modelling different disease conditions, including cystic fibrosis, infectious diseases and cancer. Cystic fibrosis was one of the first conditions to be modelled; organoids from cystic fibrosis patient ASCs (intestinal, lung and tubuloids), as well as human PSCs have been established (18,33,34,53,83). These organoids have facilitated the assessment of the cystic fibrosis transmembrane conductance regulator (CFTR) function in individual patients *in vitro*, and in turn the effect of different drugs used to treat cystic fibrosis, including VX-809 (lumacaftor) and VX-770 (ivacaftor) (34). Since CFTR mutations together with the patient’s genetic background are important host factors that contribute towards the patient’s response to therapy, *in vitro* testing in organoids has proved to be very useful as they retain these host characteristics (84). Positive correlations of *in vitro* results with clinical data strongly suggest that this approach could be used to identify treatments that will be more beneficial to patients, and at the same time more cost-efficient (39,84).

There have also been several advances in the use of organoids for drug screening for cancer therapies. Cancer organoids or tumoroids have been derived from several primary tumour types from which they retain the genetic and morphologic features (37,45,70). Organoids derived from colorectal cancer patients were used in a proof-of-concept drug screening in which drug sensitivity and its correlation with the genetic background of the organoids were investigated (37). This screening generated thousands of organoid-drug interactions and showed a wide range of sensitivities to the compound library, which included drugs in clinical use, those currently in clinical trials and those under pre-clinical investigation. They have been tested in organoids from different patients and in multiple organoids derived from the same patient (37). Tumour heterogeneity has also been addressed with organoids from other cancer types, including pancreatic ductal adenocarcinoma and liver cancer; different combinations of drugs were used to treat organoids from different patients, resulting in the organoids displaying differential responses (36,70). Liver tumoroids derived from different sections of the same primary tumour showed heterogeneity as some drugs, e.g. belinostat, dasatinib, gemcitabine and ceritinib, were effective only in a subset of the organoids (36). Due to the varied responses seen between patient organoids, this model could aid the field of personalised medicine as patient-derived organoids can be used to test drug efficacy *in vitro*, and toxicity comparisons between disease and normal organoids could identify therapies with higher efficiency and fewer side effects (45).

### Environmental toxicology

Thus far studies investigating the effects of environmental agents in organoids are scarce, although studies using both human and mouse organoids have been reported. In one study, mouse intestinal crypt organoids were exposed to cisplatin, 5-fluorouracil, ultraviolet (UV) and X-ray radiation to examine cell death and survival of intestinal epithelial cells, as well as investigate the role of certain genes in cell death regulation (42). The results obtained were closer to those obtained in primary tissue cells and *in vivo* than those obtained from immortalised cell lines, thereby demonstrating the usefulness of this model (42). Mammary organoids derived from mice have been used to study the effect of bisphenols and phthalates on the proteome (85), while human mammary organoids were recently utilised to test the effects of cadmium exposure on stem cell proliferation and differentiation by looking at organoid formation and morphology, showing a negative impact on these processes at concentrations relevant to human physiology (86). The treatment of human liver and heart organoids with lead, mercury, thallium and glyphosate led to damaging and toxic effects, such as a decrease in the beating activity of cardiac organoids (15). Human colon organoids have been used to explore the effect of prolonged ethanol exposure in healthy colon cells by analysing the changes in gene expression and chromatin accessibility, identifying almost 2000 gene expression changes (87). Others recently established an organoid-based model in which the carcinogenicity of environmental chemicals could be studied by treating organoids *in vitro* and then injecting them into nude mice (16). This study showed that the morphological carcinogenic alterations seen in the treated organoids were then also found in the nude mouse model (16). Furthermore, data from our group show that pancreatic and gastric organoids are capable of forming pre-mutagenic DNA adducts after exposure to BaP indicating that normal human tissue organoids could be useful models for genotoxicity assessment (Caipa Garcia *et al.*, unpublished results). Collectively, these first advances in the use of organoids in field of environmental and genetic toxicology indicate that organoids will allow the investigation of the relationship between the effects of environmental exposure and the increased risk of developing adverse human effects, including cancer (87). Organoid models will also allow the study of early molecular events in tumour formation and, therefore, aid the study of the modes of action and mechanisms of different carcinogens (16,87).

## Other 3D Culture Models in Toxicology

Various other 3D cell culture models have been used recently in toxicology studies which, like organoids, aim to provide a more representative microenvironment and physiology than monolayer cultures (88–90). For example, genotoxicity assays using 3D skin models have been established and validated, while liver and lung tissue models are at earlier validation stages as more robust protocols are required (91).

Spheroids, which are cellular aggregates made from cell lines, have been used in toxicological studies for different endpoints, including drug toxicity, cytotoxicity and genotoxicity (92–97). For example, liver spheroids from both primary hepatocyte (HepaRG) and tumour (HepG2, JHH1 and Huh7) cell lines have been particularly useful in the modelling of human toxicity. Multiple studies have demonstrated different cellular responses between liver, lung, bladder and mammary cell line spheroids and their monolayer cell line counterparts, showing that 3D structures are more sensitive to damage and present higher expression levels of metabolic enzymes like CYPs (92,93,95,98,99). Although spheroids provide more relevant results due to their 3D microenvironment and are relatively easy to use, they are less complex than organoids as they only contain a single cell type and are unable to replicate the relevant tissue structure (90).

Mini organ cultures (MOCs) have also been employed in toxicological studies. These cultures consist of small tissue fragments that maintain the structure of the tissues of origin and, in the case of those derived from the respiratory tract, ciliary beat activity (100–102). Although MOCs provide the 3D structure of the tissue of origin, they need to be kept at a certain size and cannot be grown for extended periods of time as their structure starts to change and viability decreases (100,101).

More complex models such as MPS have also been developed. These MPS models aim to recreate human physiological systems *in vitro* by interconnecting multiple organs in the form of organ-on-chips and/or organoids (3). MPS for many organs including the liver, heart, kidney, skeletal muscle and vasculature have been established, and these MPS organs can be connected in different combinations from 2 to 13 organs in one system depending on the interactions to be replicated (3). These systems have been used for the assessment of toxicity of drugs and other xenobiotics, including environmental compounds (103–106). Although it has been demonstrated that MPS are useful in the study of organ–organ interactions and are able to replicate a range of functions such as absorption, metabolism and contractile forces, their complexity limits the development of high throughput assays as there can be too much variability (3).

Lastly, the use of 3D *in vitro* skin models has been key in various industries to test the toxicity of compounds such as drugs and cosmetics (107). There are several skin models available, which provide different advantages depending on their origin. Due to the increase in regulations on the use of animals and the limited availability of human skin for *ex vivo* assays, artificial models made from different polymers or skin substitutes such as reconstructed epidermis or full thickness skin have become more widely used (107–109). Reconstructed skin models have been developed for normal and diseased skin, and consist of layers of human cells grown on a polymer matrix and can be of various complexities (107,110). Many different reconstructed epidermis models are commercially available and are being used to test toxicity and irritation, as well as effects of formulations (110). It has been shown that human reconstructed skin and full thickness models express XMEs although at levels that do not replicate those in native tissue (111). CYP enzyme levels are also very low in human skin and 3D skin models, but higher than in immortalised keratinocytes, so the reconstructed skin model is the better option for toxicology studies (112). Due to the ban in animal testing of cosmetics, reconstructed human skin models have been validated and are widely used; however, they are still being improved, e.g. by adding additional layers of complexity like immune cells (109,113–115).

## Conclusions

The recent progress of 3D cell culture technology has allowed the development of several assays for the study of organ development and disease, as well as toxicology and drug screens. Different 3D models of various complexities have been established, offering a more biologically relevant environment and results more representative of human physiology. Organoids have become a popular cell culture system in many fields, as they can be easily derived from stem cells (ASCs or PSCs) and retain some of the cell types, structure and function of the organ of origin. Organoids from different tissues have been established from both ASCs and PSCs, with organoids derived from PSCs being slightly more complex than those derived from ASCs.

Organoids have great potential in the study of organ development, disease modelling, drug development and organ regeneration. It has been shown that organoids from different tissues express functional XMEs including CYPs, such as CYP3A4, CYP1A1, CYP1A2 and CYP3A11. Drug development has benefitted from this as organoids have facilitated pre-clinical testing of more personalised treatments and large screens for efficacy and toxicity of a range of compounds.

Although the use of organoids in environmental and genetic toxicology has been more limited, the treatment of organoids with various environmental compounds has generated results close to those previously obtained in primary tissue and *in vivo* models, demonstrating the biological relevance of this model. Other studies have investigated the toxicity of some compounds on different tissue organoids, providing promising results that indicate organoids will facilitate the study of the effects of environmental exposure and the increased risk of developing diseases like cancer. More in-depth analyses of the metabolic capabilities of organoids would help to validate the use of this system to investigate the modes of action of environmental carcinogens. Organoids show great potential for environmental and genetic toxicology research and they will allow us to expand our knowledge of the molecular mechanisms of carcinogenesis, contributing to risk assessment. Initially such advances are likely to emerge from bespoke investigations of the mechanisms of action of specific agents in target organs. Current barriers to their more widespread use, e.g. in routine safety testing are the cost and complexity of organoid culture. Establishing organoid ‘lines’ with stable karyotypes and prolonged growth potential will also be required in the longer term.
